# Fine Mapping and Candidate Gene Search of Quantitative Trait Loci for Growth and Obesity Using Mouse Intersubspecific Subcongenic Intercrosses and Exome Sequencing

**DOI:** 10.1371/journal.pone.0113233

**Published:** 2014-11-14

**Authors:** Akira Ishikawa, Sin-ichiro Okuno

**Affiliations:** Laboratory of Animal Genetics, Graduate School of Bioagricultural Sciences, Nagoya University, Nagoya, Aichi, Japan; CSIRO, Australia

## Abstract

Although growth and body composition traits are quantitative traits of medical and agricultural importance, the genetic and molecular basis of those traits remains elusive. Our previous genome-wide quantitative trait locus (QTL) analyses in an intersubspecific backcross population between C57BL/6JJcl (B6) and wild *Mus musculus castaneus* mice revealed a major growth QTL (named *Pbwg1*) on a proximal region of mouse chromosome 2. Using the B6.Cg-*Pbwg1* intersubspecific congenic strain created, we revealed 12 closely linked QTLs for body weight and body composition traits on an approximately 44.1-Mb wild-derived congenic region. In this study, we narrowed down genomic regions harboring three (*Pbwg1.12*, *Pbwg1.3* and *Pbwg1.5*) of the 12 linked QTLs and searched for possible candidate genes for the QTLs. By phenotypic analyses of F_2_ intercross populations between B6 and each of four B6.Cg-*Pbwg1* subcongenic strains with overlapping and non-overlapping introgressed regions, we physically defined *Pbwg1.12* affecting body weight to a 3.8-Mb interval (61.5–65.3 Mb) on chromosome 2. We fine-mapped *Pbwg1.3* for body length to an 8.0-Mb interval (57.3–65.3) and *Pbwg1.5* for abdominal white fat weight to a 2.1-Mb interval (59.4–61.5). The wild-derived allele at *Pbwg1.12* and *Pbwg1.3* uniquely increased body weight and length despite the fact that the wild mouse has a smaller body size than that of B6, whereas it decreased fat weight at *Pbwg1.5*. Exome sequencing and candidate gene prioritization suggested that *Gcg* and *Grb14* are putative candidate genes for *Pbwg1.12* and that *Ly75* and *Itgb6* are putative candidate genes for *Pbwg1.5*. These genes had nonsynonymous SNPs, but the SNPs were predicted to be not harmful to protein functions. These results provide information helpful to identify wild-derived quantitative trait genes causing enhanced growth and resistance to obesity.

## Introduction

Body weight and body composition traits, including fat and organ weight, are quantitative in nature and are controlled by multiple genetic loci, referred to as QTLs (quantitative trait loci), environmental factors and their interactions. They are important economic traits in livestock [1]. For example, modern broiler chickens have been intensively selected for rapid growth rate, but they display excessive deposition of body fat. Since fat is a by-product of little economic value and often causes a decrease in feed efficiency, it is now an important selection criterion in chicken breeding programs [2]. In humans, obesity is characterized by excessive abdominal fat deposition and is now a main health concern worldwide because it is a predisposing factor of complex metabolic diseases such as type-2 diabetes and cardiovascular diseases [3]. The laboratory mouse has been long and widely used as the premier model animal for elucidating the genetic and molecular basis of these traits and other quantitative traits in livestock and humans because of its small body size, cost-effective rearing, easy development of genetically engineered mice (e.g., knockouts and transgenics) and large amount of genomic information that is freely available [1], [4]. Thousands of QTLs affecting various quantitative traits have been mapped to many chromosomal regions of the mouse and have been deposited in the Mouse Genome Database (MGD, release 5.19, August 2014) [5].

However, the genetic and molecular basis of quantitative traits remains elusive because it is not an easy task to pinpoint causative genes underlying QTLs, particularly for QTLs with small phenotypic effects on the traits. Most of the QTLs have small effects and only a few loci have moderate to large effects [4]. In mice, initial genome-wide QTL analysis is usually performed with a backcross or F_2_ intercross population between two inbred strains and it provides a large confidence interval (10–50 cM) for a mapped QTL [6], where hundreds or thousands of genes are possibly located. Next, to reduce the confidence interval of the QTL to a level amenable to positional cloning, fine mapping is performed using a congenic mouse strain and subsequently developed subcongenic strains [7]. Then phenotypic values are compared between homozygous congenic/subcongenic strains and the background strain and/or among homozygous congenic/subcongenic strains with overlapping and non-overlapping introgressed regions. Often, the phenotypic effect of the QTL fails to be confirmed, illustrating the difficulty of identifying a causative gene for the QTL. If the QTL successfully is fine-mapped to a small region, the road from a QTL to a causative gene is still long [8]. In the present study, to overcome the problem of frequent failure in traditional congenic/subcongenic analyses, we used several F_2_ populations obtained from intercrosses between each of the subcongenic strains and the background strain. In each of the F_2_ populations, three possible diplotypes for the introgressed region are segregating: two are homozygous for either haplotype derived from the donor mouse or from the background mouse and the other is heterozygous for both haplotypes. Hence, using the F_2_ mice can randomize environmental effects such as litter size and effects of contaminating donor and recipient alleles on unwanted small regions, both of which are produced by double recombination during recurrent backcrossing for development of subcongenic strains, as previously documented [9], [10]. Moreover, the F_2_ mice produced have genetically identical F_1_ dams and F_1_ sires. That is, the F_1_ mice are heterozygotes for all loci on the introgressed region. Hence, using the F_2_ mice can minimize genomic imprinting effects of alleles inherited from either F_1_ dams or F_1_ sires [11] and maternal genetic effects exerted from the F_1_ dams [12] and epigenetic effects such as histone modification [13]–[15] exerted from either or both F_1_ parents. Probably, some of these effects result in the missing QTL effect seen in traditional congenic/subcongenic analyses.

We previously discovered many QTLs for postnatal body weight and growth from an untapped resource of wild *M. m. castaneus* mice captured live in the Philippines, by genome-wide QTL analyses in an intersubspecific backcross population between C57BL/6JJcl (B6) inbred mice and the wild *castaneus* mice [16]–[18]. We further created the B6.Cg-*Pbwg1* congenic strain on the B6 genetic background with an approximately 44.1-Mb wild-derived genomic region between *D2Mit33* and *D2Mit38* microsatellite markers, on which *Pbwg1*, a prominent growth QTL on a proximal region of mouse chromosome 2, is located. We developed more than 20 subcongenic strains derived from B6.Cg-*Pbwg1*. By phenotypic analysis of the F_2_ intercross between B6.Cg-*Pbwg1* and B6 strains and by congenic/subcongenic analyses, we revealed 12 closely linked QTLs for body weight and body composition traits within the 44.1-Mb congenic region. These linked QTLs explained a small fraction of phenotypic variances [9], [19]–[22]. Among the linked loci, several have unique QTL effects and are located on the distal half of the congenic region. For example, the wild-derived allele at *Pbwg1.12* and *Pbwg1.3* QTLs increases body weight and total body length, respectively, despite the fact that wild mice have a smaller body size than that of B6 [19], [9]. In contrast, the allele at the *Pbwg1.5* QTL decreases abdominal white fat weight [19] and prevents obesity in mice fed both standard and high-fat diets [21].

In this study, we fine-mapped the three unique QTLs (*Pbwg1.12*, *Pbwg1.3* and *Pbwg1.5*) mentioned above by phenotypic analysis of F_2_ mice obtained from intersubspecific subcongenic intercrosses. To search for possible candidate genes of the QTLs, we performed exome sequencing of genes on the congenic region and also prioritized candidate genes using bioinformatics analysis. Sequence data are not available for our wild *M. m. castaneus* mice captured in the Philippines, in contrast to the CAST/Eij inbred strain derived from wild *M. m. castaneus* mice trapped in Thailand, for which the whole genome has been already sequenced [23].

## Materials and Methods

### Ethics Statement

This study was carried out in accordance with the guidelines for the care and use of laboratory animals of the Graduate School of Bioagricultural Sciences, Nagoya University, Japan. The protocol was approved by the Animal Research Committee of Nagoya University.

### Animals

**Figure 1:**
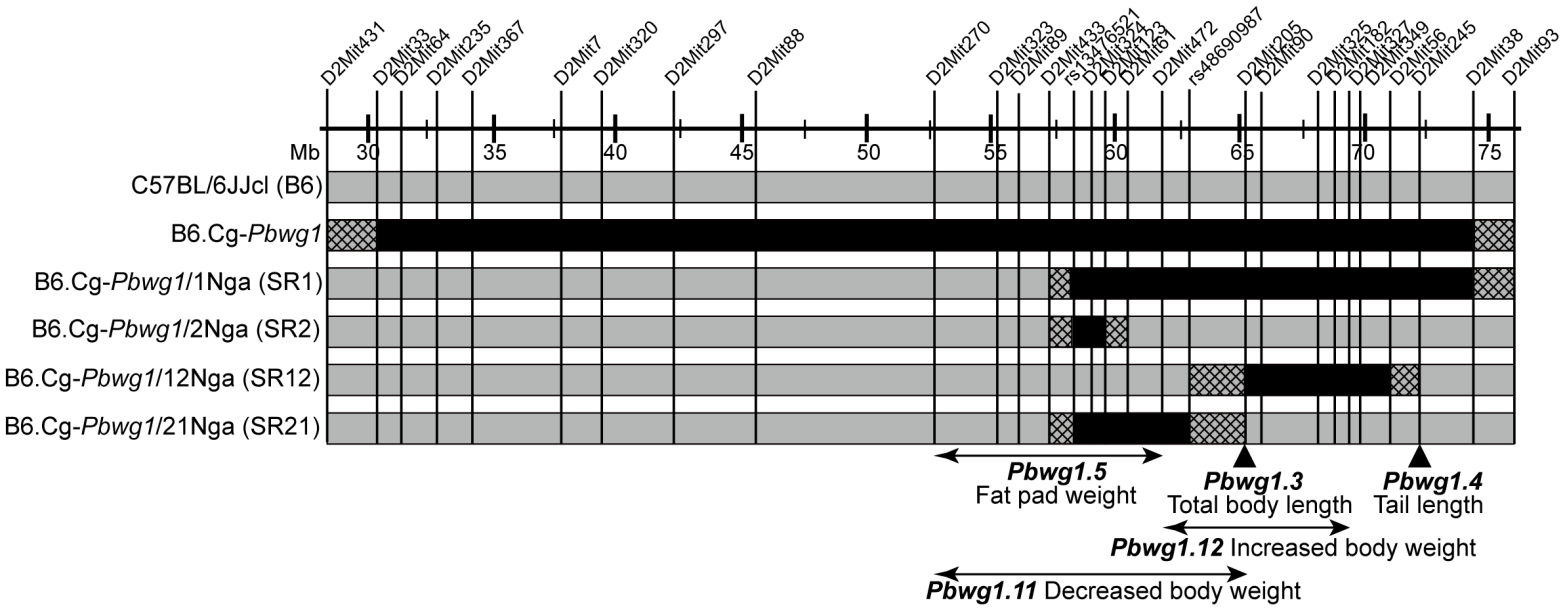
Relative introgressed genomic intervals of four subcongenic strains (B6.Cg-*Pbwg1*/#Nga, abbreviation SR#) developed from the original B6.Cg-*Pbwg1* congenic strain carrying the *Pbwg1* growth QTL on mouse chromosome 2. The black bar indicates the minimum introgressed interval derived from the wild *Mus musculus castaneus* mouse, and the gray bar indicates the interval from the background C57BL/6JJcl (B6) strain. The hatched bar shows a gray zone where recombination occurred. The physical map positions (Mb) of 27 microsatellite markers (*D2Mit#*) and two PCR-RFLP markers (*rs#*) developed in this study (Figure S1) are shown on the horizontal line. The triangle indicates the position of the peak LOD score for three body composition QTLs (*Pbwg1.3* to *Pbwg1.5*) previously identified [19], [21]. The horizontal double-headed arrows indicate the maximum intervals of two body weight QTLs (*Pbwg1.11* and *Pbwg1.12*) previously defined [9].

The B6.Cg-*Pbwg1* congenic strain was previously constructed [19]. Many subcongenic strains, named B6.Cg-*Pbwg1*/#Nga (old name: B6.Cg-*Pbwg1*/SR#, called SR# hereafter), were previously developed from descendants of B6.Cg-*Pbwg1* [9]. Previously developed SR1, SR3 and SR12 subcongenic strains and a newly developed SR21 subcongenic strain were used in this study (Figure 1). The background C57BL/6JJcl (B6) mice were purchased from Clea Japan (Tokyo, Japan). To develop four F_2_ segregating populations, males of each subcongenic strain were crossed with B6 females to generate F_1_ mice. The F_1_ mice obtained were mated *inter se*. In total, the following F_2_ individuals were produced: 273 (138 males and 135 females) for B6×SR1, 236 (113 males and 123 females) for B6×SR2, 132 (58 males and 74 females) for B6×SR12 and 291 (151 males and 140 females) for B6×SR21. Litter size was not standardized at birth to maximize the number of F_2_ mice reared.

All mice were weaned at 3 weeks of age. Littermates of the same sex were housed in groups of up to four mice per cage. Standard chow (CA-1, Clea Japan), containing 27% crude protein, 5% crude fat, 3% crude fiber, 8% crude ash and 3.5 kcal/g, and tap water were provided *ad libitum*. The mice were reared in an environment with a temperature of 23±3°C, 55% relative humidity, and a light/dark cycle of 12∶12.

### Genotyping

Genomic DNAs were extracted with a standard method from ear clips of the F_2_ mice. Microsatellite markers located within each of the subcongenic intervals (Figure 1) were genotyped as described previously [19]. To reduce as short as possible the gray regions flanking the subcongenic intervals, where recombination occurred, we newly developed two PCR-RFLP markers based on two SNPs identified by exome sequencing in this study (Figure S1). Each of the F_2_ mice had one of three diplotypes (B/B, B/C and C/C), where B is the haplotype derived from B6 mice and C is the haplotype derived from wild *castaneus* mice. Diplotype configuration was determined for each mouse of the four F_2_ populations produced. F_2_ mice having recombination within the subcongenic interval were excluded from this study.

### Phenotyping

Body weight of F_2_ mice was measured to the nearest 0.01 g at 1, 3, 6, 10 and 14 weeks of age. Four body weight gains at 1–3 weeks, 3–6 weeks, 6–10 weeks and 10–14 weeks of age were calculated. After overnight fasting, mice were sacrificed under anesthesia. Total body length (from the tip of the nose to the end of the tail) and tail length (from the anus to the end of the tail) were immediately measured to the nearest 0.01 cm. Head-body length was obtained by subtracting tail length from total body length. After taking a blood sample by cardiopuncture, the lungs, spleen, liver, kidneys and testes were dissected and weighed to the nearest 0.001 g. In addition, the weights of two-sided inguinal and gonadal (epidydimal in males and parametrial in females) fat pads were recorded. In mice, the weight of white fat depots such as gonadal fat pads has been long and widely used as an indicator of fatness because the fat depots can be easily dissected out and are highly correlated with total body fat [1].

Body weight, weight gain and body composition data obtained for the F_2_ populations were analyzed with a linear mixed model of the statistical discovery software JMP version 11.1.1 (SAS Institute, Cary, NC) in which diplotype, sex, parity, litter size and their possible two-way interactions were treated as fixed effects and dam was treated as a random effect. The fixed effects and interaction effects that were significant at the nominal 5% level were included in the final model. Phenotypic differences among diplotypes were determined by one-way analysis of variance followed by Tukey's HSD test. To adjust for multiple testing, Bonferroni-corrected 5% level was finally used as a significant threshold.

To estimate additive and dominance effects of diplotypes, a linear mixed model was fitted, with additive, dominance, sex, parity, litter size and their possible two-way interactions being included as fixed effects and dam being included as a random effect. The fixed effects and interaction effects that were significant at the nominal 5% level were finally included in the model. As previously defined for allelic effects of a QTL [24], additive diplotype effect is half of the difference between B/B and C/C homozygous diplotypes, and dominance diplotype effect is the difference between B/C heterozygous diplotypes and the average of B/B and C/C homozygous diplotypes. To estimate the additive diplotype effect, diplotypes were assigned quantitatively as −1 for B/B homozygotes, zero for B/C heterozygotes and +1 for C/C homozygotes. To estimate the dominance diplotype effect, diplotypes were assigned as zero for two types of homozygotes and +1 for heterozygotes. The degree of dominance was calculated as the ratio of dominance diplotype effect to additive diplotype effect.

### Exome Sequencing

Genomic DNA was extracted with a standard method from the tail of a B6.Cg-*Pbwg1* congenic mouse. Enrichment of exon regions in the 44.1-Mb congenic interval on mouse chromosome 2 was performed using Roche NimbleGen sequence capture arrays that were custom-made on the basis of UCSC Mouse Genome Browser NCBI37/mm9 assembly (RefSeq mm9). Enrichment experiments and exome sequencing with the next-generation sequencer Roche GS FLX were outsourced to Hokkaido System Science Co., Ltd (Sapporo, Japan). Sequence reads obtained were mapped to RefSeq mm9, and then synonymous SNPs (sSNPs), nonsynonymous SNPs (nsSNPs), indels (insertions and deletions) and nonsense mutations were investigated.

### Candidate Gene Search

Endevour is a web-based computational software program that prioritizes candidate genes with respect to their biological processes or diseases of interest [25]. Genes on target regions carrying QTLs for body weight and obesity were prioritized on the basis of similarity to training genes that have already been shown to be involved in body weight and obesity regulation (Table S1). The training genes used were searched using Online Mendelian Inheritance in Man (OMIM) database (http://www.omim.org).

Effects of nsSNPs identified by exome sequencing on protein functions were investigated with two web-based software programs, SIFT [26] and PolyPhen-2 [27]. SIFT predicts tolerated and deleterious substitutions for nsSNPs based on the evolutionary conservation of amino acids within protein families [26]. PolyPhen-2 predicts possible impact of an amino acid substitution on the structure and function of a protein using straightforward physical and comparative considerations [27]. Since PolyPhen-2 was developed for human proteins, this software was implemented after converting the positions of amino acid substitutions in our mouse study to the corresponding positions of the human protein.

## Results

### Intersubspecific Subcongenic Intercross Analyses

Most growth and body composition traits examined in F_2_ segregating populations between each of the four subcongenic strains (Figure 1) and the background B6 strain showed significant interactions between sex and trait (data not shown). We thus performed statistical comparisons of these traits among mice with three diplotypes in each sex separately.

**Figure 2:**
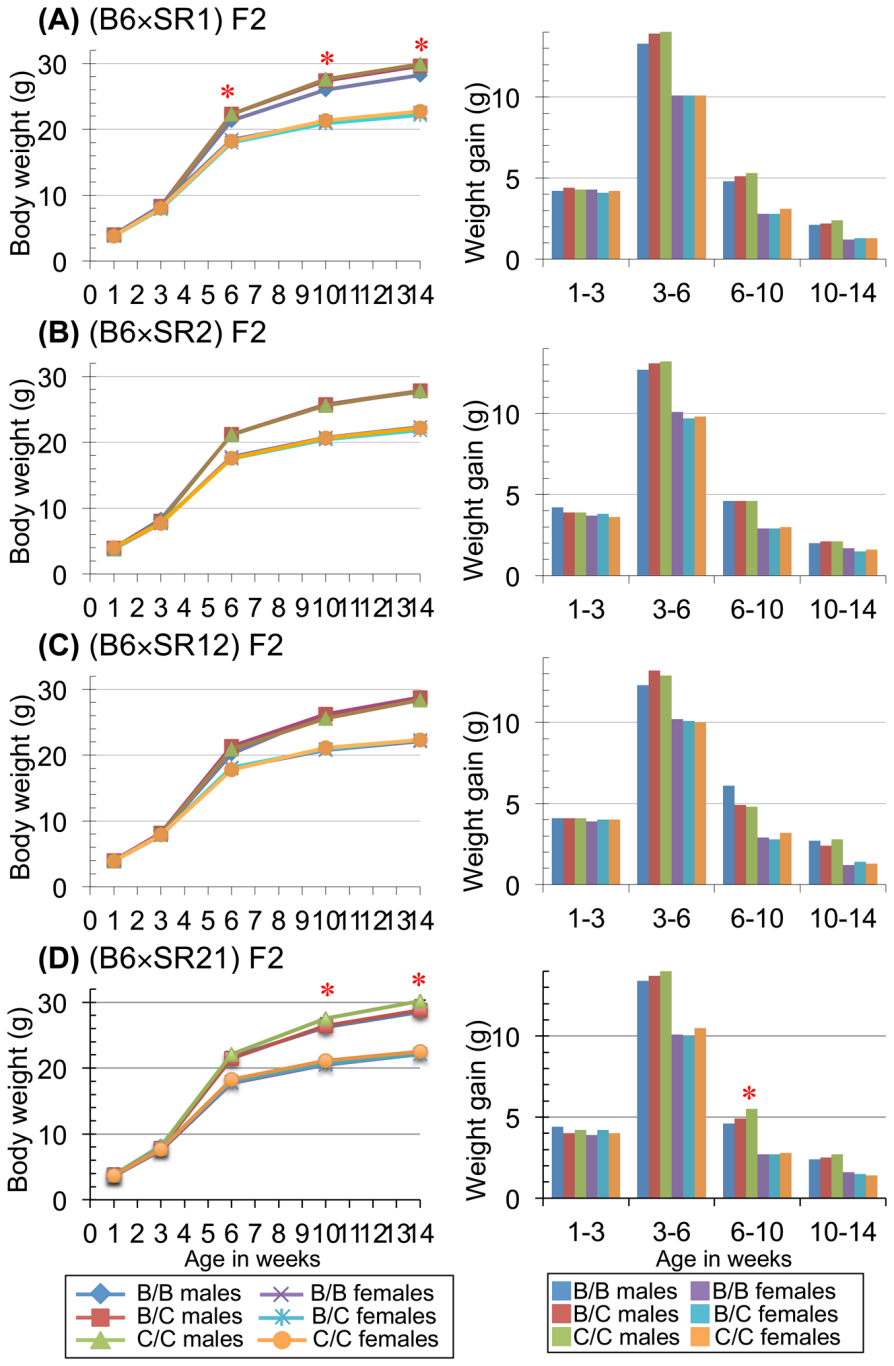
Comparisons of body weight and body weight gain among three diplotypes (B/B, B/C and C/C) in four F_2_ segregating populations obtained from (A) B6×SR1, (B) B6×SR2, (C) B6×SR12 and (D) B6×SR21 intercrosses. B indicates the haplotype derived from B6 mice and C indicates the haplotype derived from wild *castaneus* mice. The asterisk shows significant differences in the trait among three diplotypes in each sex and the *P* values exceeded the Bonferroni-corrected 5% levels (see text for details).

Figure 2 shows measurements of body weight and body weight gain in the four F_2_ segregating populations. In the B6×SR1 intercross, body weight of male mice with C/C diplotypes at 6 weeks of age was significantly higher than that of mice with the B/B diplotype (*P* = 0.0041, Tukey's HSD test) at the Bonferroni-corrected 5% significance level. However, it was not different from that of B/C males throughout ages examined. From 6 weeks onwards, the weight difference between C/C and B/B males remained significant (*P* = 0.0000036 at 10 weeks and 0.0000017 at 14 weeks of age). Additive diplotype effects for body weights at 6, 10 and 14 weeks of age exceeded the Bonferroni-corrected 5% level, whereas dominance diplotype effects were not significantly different from zero (Table 1). The C haplotype derived from wild mice increased body weight, despite the fact that the wild mice have approximately 60% of the body weight of B6 mice [16]. The mode of inheritance of this haplotype was additive or dominant (Table 1). In females, however, there were no significant differences in body weight at any age (Figure 2). For body weight gains at 1–3 weeks, 3–6 weeks, 6–10 weeks and 10–14 weeks, both sexes of mice with three diplotypes did not show significant differences at the Bonferroni-corrected 5% level. Although body weight gain in males at 3–6 weeks (*P* = 0.0058) was on the border of that level, its additive diplotype effect surpassed it (Table 1).

**Table 1:** Additive and dominance diplotype effects for body weight at # weeks of age and weight gain between # and # weeks of age in the F_2_ populations obtained from B6×SR1 and B6×SR21 intercrosses.

			Diplotype effect^a^					
Sex	F_2_ population	Trait	Additive	*P* value	Dominance	*P* value	Degree of dominance	Inheritance^b^
Male	B6×SR1	Wt1	0.039±0.046	0.40	0.049±0.069	0.47	-	-
		Wt3	0.041±0.111	0.71	0.193±0.170	0.26	-	-
		Wt6	0.525±0.173	**0.0030**	0.376±0.264	0.16	0.72	**Add**
		Wt10	0.788±0.167	**0.0000076**	0.582±0.256	0.025	0.73	**Dom**
		Wt14	0.874±0.178	**0.0000034**	0.586±0.271	0.033	0.67	**Dom**
		Gain1–3	0.023±0.095	0.81	0.151±0.142	0.29	-	-
		Gain3–6	0.395±0.128	**0.0026**	0.206±0.196	0.30	0.52	Add
		Gain6–10	0.267±0.123	0.031	0.040±0.185	0.83	0.15	Add
		Gain10–14	0.160±0.102	0.12	−0.021±0.153	0.89	-	-
	B6×SR21	Wt1	−0.001±0.043	0.98	−0.034±0.062	0.59	-	-
		Wt3	−0.066±0.097	0.49	−0.319±0.140	0.024	−4.83	Overrec
		Wt6	0.198±0.164	0.23	−0.379±0.238	0.11	-	-
		Wt10	0.635±0.172	**0.00032**	−0.483±0.249	0.055	−0.76	**Add**
		Wt14	0.833±0.192	**0.000030**	−0.552±0.279	0.050	−0.66	**Rec**
		Gain1–3	−0.074±0.070	0.29	−0.282±0.102	0.0065	−3.81	Overrec
		Gain3–6	0.277±0.144	0.016	−0.045±0.166	0.78	−0.16	Add
		Gain6–10	0.440±0.121	**0.00039**	−0.099±0.176	0.57	−0.23	**Add**
		Gain10–14	0.155±0.072	0.033	−0.080±0.105	0.45	−0.52	Add
Female	B6×SR1	Wt1	−0.069±0.047	0.15	−0.146±0.063	0.023	−2.12	Overrec
		Wt3	−0.156±0.091	0.089	−0.278±0.120	0.023	−1.78	Overrec
		Wt6	0.006±0.136	0.97	−0.092±0.185	0.62	-	-
		Wt10	0.060±0.122	0.62	−0.423±0.164	0.011	−7.05	Overrec
		Wt14	0.099±0.136	0.47	−0.379±0.182	0.040	−3.83	Overrec
		Gain1–3	−0.079±0.061	0.19	−0.160±0.081	0.051	−2.03	Overrec
		Gain3–6	0.010±0.111	0.93	−0.030±0.152	0.84	-	-
		Gain6–10	0.177±0.096	0.069	−0.143±0.130	0.27	-	-
		Gain10–14	0.033±0.079	0.68	0.058±0.107	0.59	-	-
	B6×SR21	Wt1	0.039±0.042	0.36	0.003±0.058	0.96	-	-
		Wt3	0.094±0.096	0.33	0.207±0.132	0.12	-	-
		Wt6	0.271±0.114	0.019	−0.103±0.157	0.51	−0.38	Add
		Wt10	0.301±0.119	0.013	−0.201±0.165	0.22	−0.67	Add
		Wt14	0.208±0.120	0.086	−0.204±0.116	0.22	-	-
		Gain1–3	0.058±0.068	0.39	0.216±0.094	0.023	3.72	Overdom
		Gain3–6	0.181±0.110	0.10	−0.287±0.152	0.061	-	-
		Gain6–10	0.027±0.091	0.77	−0.097±0.127	0.44	-	-
		Gain10–14	−0.088±0.080	0.27	−0.006±0.111	0.96	-	-

Data are means and standard errors. The *P* value in bold exceeded the Bonferroni-corrected 5% threshold level.

a Positive sign indicates that the haplotype derived form the wild *castaneus* mouse increased the trait value.

b Add, additive; Dom, dominant; Rec, recessive; Overrec, overrecessive; Overdom, overdominant; -, not applicable.

In B6×SR2 and B6×SR12 intercrosses, body weights at any age did not differ significantly among mice with three diplotypes in both sexes. Similarly, there were no significant diplotype differences in body weight gains at any age in both sexes (Figure 2 and Table S2).

On the other hand, in the B6×SR21 intercross, body weights of C/C males were significantly higher than those of B/C and B/B males at 10 weeks (*P* = 0.00028) and 14 weeks (*P* = 0.000029) of age (Figure 2). The wild-derived C haplotype was inherited in an additive or recessive fashion (Table 1). Furthermore, body weight gain at 6–10 weeks was significantly higher in the C/C males than in the B/C and B/B males (*P* = 0.0016) (Figure 2). The C haplotype was inherited in an additive fashion (Table 1). In contrast, neither body weight nor body weight gain was significantly different among females with three diplotypes at the Bonferroni-corrected 5% level (Figure 2 and Table 1).

Tables 2 and S3 show measurements of body composition traits not adjusted for final body weight at 14 weeks of age in two kinds of F_2_ populations obtained from B6×SR1 and B6×SR21 intercrosses. In the B6×SR1 intercross, head-body length and total body length of C/C males were both significantly larger than those of B/B males at the Bonferroni-corrected 5% level (Table 2). The same tendency was observed for those traits in females, but the C haplotype was transmitted as different modes of inheritance in males (additive) and females (overrecessive) for an unknown reason. For inguinal fat pad weight and gonadal fat pad weight, C/C mice had the lowest values among mice with three diplotypes in both sexes. The C haplotype was inherited in an additive or recessive fashion depending on the sex. A significant difference in kidney weight was observed only in females (Table S3).

**Table 2:** Body length and fat pad weight not adjusted for body weight at 14 weeks of age, and additive and dominance diplotype effects for body length and fat pad weight in the F_2_ populations obtained from B6×SR1 and B6×SR21 intercrosses.

			Diplotype^c^				Diplotype effect				Degree of dominance	
Sex	F_2_ population	Trait	B/B	B/C	C/C	*P* value	Additive	*P* value	Dominance	*P* value		Inheritance^d^
Male	B6×SR1	No. of mice	33	33–34	35–37							
		Tail length (cm)	8.33±0.04	8.42±0.04	8.39±0.04	0.13	0.028±0.024	0.24	0.060±0.038	0.12	-	-
		Head-body length (cm)	9.20±0.04^a^	9.34±0.04^b^	9.38±0.04^b^	**0.00079**	0.088±0.025	**0.00080**	0.058±0.041	0.17	0.66	**Add**
		Total body length (cm)	17.52±0.06^a^	17.76±0.06^b^	17.77±0.06^b^	**0.0014**	0.118±0.038	**0.0028**	0.116±0.063	0.070	0.98	**Add**
		Inguinal fat pad weight (g)	0.278±0.012^a^	0.257±0.012^a^	0.203±0.012^b^	**0.0000069**	−0.037±0.012	**0.0000035**	0.016±0.012	0.17	0.43	**Add**
		Gonadal fat pad weight (g)	0.280±0.014^a^	0.275±0.014^a^	0.210±0.014^b^	**0.000038**	−0.034±0.008	**0.000071**	0.030±0.013	0.030	0.88	**Rec**
	B6×SR21	No. of mice	32	38–39	38–39							
		Tail length (cm)	8.38±0.04	8.38±0.03	8.45±0.03	0.13	0.034±0.021	0.11	−0.039±0.033	0.24	-	-
		Head-body length (cm)	9.26±0.04^a^	9.27±0.03^a^	9.41±0.03^b^	**0.00021**	0.078±0.021	**0.00035**	−0.070±0.033	0.038	−0.90	**Rec**
		Total body length (cm)	17.62±0.05^a^	17.63±0.05^a^	17.85±0.06^b^	**0.00022**	0.115±0.032	**0.00044**	−0.110±0.050	0.032	−0.96	**Rec**
		Inguinal fat pad weight (g)	0.384±0.016	0.382±0.015	0.404±0.015	0.35	0.010±0.009	0.25	−0.012±0.014	0.39	-	-
		Gonadal fat pad weight (g)	0.362±0.016	0.362±0.015	0.398±0.015	0.066	0.018±0.009	0.051	−0.018±0.014	0.210	-	-
Female	B6×SR1	No. of mice	24	34–36	32							
		Tail length (cm)	8.14±0.03^ab^	8.11±0.03^b^	8.20±0.03^a^	0.038	0.030±0.021	0.16	−0.063±0.031	0.048	−2.10	Overrec
		Head-body length (cm)	8.88±0.05^ab^	8.77±0.04^b^	8.91±0.04^a^	0.023	0.013±0.030	0.67	−0.122±0.045	0.0081	−9.38	Overrec
		Total body length (cm)	17.02±0.06^ab^	16.89±0.05^b^	17.12±0.06^a^	**0.0043**	0.049±0.039	0.22	−0.178±0.059	**0.0034**	−3.63	**Overrec**
		Inguinal fat pad weight (g)	0.231±0.13^a^	0.193±0.012^b^	0.183±0.012^b^	**0.0037**	−0.024±0.007	**0.0013**	−0.015±0.011	0.17	−0.63	**Add**
		Gonadal fat pad weight (g)	0.176±0.012^a^	0.137±0.010^b^	0.133±0.010^b^	**0.0019**	−0.022±0.006	**0.0011**	−0.018±0.009	0.065	−0.82	**Add**
	B6×SR21	No. of mice	28	38–39	33							
		Tail length (cm)	8.00±0.03^a^	8.09±0.03^ab^	8.16±0.03^b^	**0.0024**	0.077±0.021	**0.00052**	0.011±0.032	0.73	0.14	**Add**
		Head-body length (cm)	8.85±0.04^ab^	8.88±0.03^b^	8.98±0.03^a^	0.012	0.065±0.023	0.0064	−0.035±0.036	0.33	−0.54	Add
		Total body length (cm)	16.85±0.06^a^	16.99±0.05^a^	17.15±0.05^b^	**0.00051**	0.150±0.004	**0.00011**	−0.013±0.057	0.82	−0.87	**Rec**
		Inguinal fat pad weight (g)	0.382±0.14^a^	0.371±0.012^ab^	0.374±0.013^b^	0.039	−0.019±0.008	0.017	0.008±0.012	0.50	0.42	Add
		Gonadal fat pad weight (g)	0.258±0.013	0.243±0.012	0.226±0.012	0.072	−0.016±0.007	0.024	0.001±0.010	0.92	0.063	Add

Trait data are means and standard errors computed using a linear model including fixed and random effects (see Materials and Methods).

a,b Means with different superscript letters within a trait indicate significant differences among three deplotypes at *P*≤0.05 (Tukey's HSD test). The *P* value in bold exceeded the Bonferroni-corrected 5% threshold level.

c B denotes the haplotype where all alleles at marker loci on the congenic region are fixed for B6 alleles, and C indicates the haplotype on which all alleles are fixed for wild-derived *castaneus* alleles. Individuals with recombinant haplotypes were excluded from this analysis.

d Add, additive; Rec, recessive; Overrec, overrecessive; -, not applicable.

In the B6×SR21 intercross, unadjusted body length traits were significantly different in both sexes at the Bonferroni-corrected 5% level. C/C mice had the longest length in both sexes (Table 2). The C/C mice had significantly higher kidney weight in males but not in females (Table S3).

On the other hand, in both B6×SR2 and B6×SR12 intercrosses, no unadjusted traits were significantly different among mice with three diplotypes at the Bonferroni-corrected 5% level as well as the nominal 5% level (Table S4).

Tables 3 and S5 show measurements of body composition traits adjusted for final body weight at 14 weeks of age, which are body-size-free traits, in two F_2_ populations obtained from B6×SR1 and B6×SR21 intercrosses. In the B6×SR1 intercross, significant diplotype differences were observed in inguinal and gonadal fat pad weights for both sexes and in testis weight for males at the Bonferroni-corrected 5% level. Both of the fat weights in C/C mice were lowest in each sex. Testis weight in C/C males was also lowest. The C haplotype for these three traits indicated an additive mode of inheritance. In contrast, in the B6×SR21 intercross, inguinal and gonadal fat pad weights were significantly lower in C/C females than in B/B females at the nominal 5% level, but those fat weights were not significantly different in males.

**Table 3:** Body length and fat pad weight adjusted for body weight at 14 weeks of age, and additive and dominance diplotype effects for body length and fat pad weight in the F_2_ populations obtained from B6×SR1 and B6×SR21 intercrosses.

			Diplotype				Diplotype effect				Degree of dominance	
Sex	F_2_ population	Trait	B/B	B/C	C/C	*P* value	Additive	*P* value	Dominance	*P* value		Inheritance
Male	B6×SR1	No. of mice	33	33–34	35–37							
		Tail length (cm)	8.37±0.04^a^	8.40±0.04^a^	8.37±0.04^a^	0.70	−0.003±0.024	0.91	0.032±0.037	0.39	-	-
		Head-body length (cm)	9.27±0.03^a^	9.30±0.03^a^	9.32±0.03^a^	0.52	0.022±0.021	0.31	0.010±0.033	0.76	-	-
		Total body length (cm)	17.64±0.05^a^	17.71±0.05^a^	17.69±0.05^a^	0.56	0.021±0.032	0.51	0.042±0.051	0.41	-	-
		Inguinal fat pad weight (g)	0.287±0.013^a^	0.249±0.012^b^	0.198±0.012^c^	**0.00000049**	−0.042±0.008	**0.00000097**	0.013±0.012	0.28	0.31	**Add**
		Gonadal fat pad weight (g)	0.290±0.014^a^	0.269±0.014^a^	0.203±0.014^b^	**0.0000028**	−0.042±0.009	**0.0000036**	0.023±0.013	0.088	0.55	**Add**
	B6×SR21	No. of mice	32	38–39	38–39							
		Tail length (cm)	8.40±0.03	8.39±0.03	8.41±0.03	0.83	0.008±0.002	0.69	−0.016±0.032	0.63	-	-
		Head-body length (cm)	9.31±0.02	9.32±0.02	9.35±0.02	0.49	0.017±0.016	0.28	−0.013±0.024	0.58	-	-
		Total body length (cm)	17.71±0.04	17.70±0.03	17.76±0.04	0.39	0.026±0.024	0.29	−0.340±0.372	0.36	-	-
		Inguinal fat pad weight (g)	0.386±0.016	0.384±0.015	0.398±0.016	0.71	0.006±0.009	0.53	−0.008±0.014	0.57	-	-
		Gonadal fat pad weight (g)	0.366±0.016	0.366±0.015	0.388±0.016	0.40	0.011±0.009	0.26	−0.011±0.014	0.42	-	-
Female	B6×SR1	No. of mice	24	34–36	32							
		Tail length (cm)	8.10±0.03^a^	8.14±0.03^a^	8.18±0.03^a^	0.096	0.027±0.018	0.14	−0.018±0.028	0.51	-	-
		Head-body length (cm)	8.86±0.03^a^	8.82±0.03^a^	8.87±0.03^a^	0.43	0.006±0.022	0.78	−0.043±0.035	0.21	-	-
		Total body length (cm)	16.96±0.040^a^	16.96±0.034^a^	17.05±0.036^a^	0.040	0.035±0.024	0.14	−0.056±0.036	0.13	-	-
		Inguinal fat pad weight (g)	0.227±0.012^a^	0.200±0.010^ab^	0.178±0.011^b^	**0.0030**	−0.025±0.007	**0.00066**	−0.003±0.011	0.82	−0.12	**Add**
		Gonadal fat pad weight (g)	0.173±0.010^a^	0.145±0.008^ab^	0.127±0.009^b^	**0.0019**	−0.021±0.006	**0.00071**	−0.003±0.009	0.71	−0.14	**Add**
	B6×SR21	No. of mice	28	38–39	33							
		Tail length (cm)	8.03±0.03^a^	8.10±0.03^ab^	8.13±0.03^b^	0.027	0.050±0.018	0.0076	0.014±0.027	0.60	-	-
		Head-body length (cm)	8.88±0.03	8.89±0.03	8.95±0.03	0.093	0.039±0.020	0.054	−0.026±0.030	0.38	-	-
		Total body length (cm)	16.91±0.047^a^	16.97±0.041^ab^	17.08±0.044^b^	0.011	0.086±0.028	**0.0035**	−0.017±0.042	0.69	−0.20	Add
		Inguinal fat pad weight (g)	0.385±0.014^a^	0.372±0.012^ab^	0.341±0.013^b^	0.016	−0.022±0.008	0.0068	0.009±0.012	0.45	-	-
		Gonadal fat pad weight (g)	0.261±0.013^a^	0.244±0.011^ab^	0.222±0.012^b^	0.021	−0.020±0.007	0.0061	0.002±0.010	0.82	-	-

For abbreviations, see the footnotes to Tables 1 and 2.

On the other hand, in B6×SR2 and B6×SR12 intercrosses, neither males nor females showed significant diplotype differences in any adjusted body composition traits at the Bonferroni-corrected 5% level (Table S6).

Taking all results together, a growth QTL, the wild-derived allele of which increased body weight and body weight gain, was localized to an interval between *D2Mit433* (57.3 Mb) and *D2Mit205* (65.3), as summarized in Table 4. In a previous study using congenic strains [9], the growth QTL *Pbwg1.12* was physically localized to a maximum interval between *D2Mit472* (61.5) and *D2Mit327* (69.5) (see Figure 1), which overlapped with the interval of the growth QTL identified in this study. The wild-derived allele at *Pbwg1.12* increased body weight [9], which was exactly the same allelic effect of the growth QTL identified in this study. Therefore, in this study, we succeeded in confirming the presence of *Pbwg1.12* and in narrowing it down to a 3.8-Mb interval between *D2Mit472* (61.5) and *D2Mit205* (65.3).

**Table 4:** Summary of QTLs for growth and body composition confirmed in the four F_2_ segregating populations.

	F_2_ population^a^				QTL	
Trait	B6×SR1	B6×SR2	B6×SR12	B6×SR21	Symbol	Genomic interval (Mb)
Body weight & weight gain	Increased	ND	ND	Increased	*Pbwg1.12* b	*D2Mit472*-*D2Mit205* (61.5–65.3)
Total body length (unadjusted)	Increased	ND	ND	Increased	*Pbwg1.3*	*D2Mit433*-*D2Mit205* (57.3–65.3)
Fat pad weight (unadjusted & adjusted)	Decreased	ND	ND	MG	*Pbwg1.5* c	*D2Mit123*-*D2Mit472* (59.4–61.5)

a The effect of the QTL allele derived from the wild mouse is shown: Increased, increased the trait value; Decreased, decreased the trait value; MG, decreased the trait value but the QTL effect was marginal; ND, QTL was not detected.

b *Pbwg1.12* was previously defined to an interval between *D2Mit472* and *D2Mit327* [9] (see Figure 1 for their relative map positions).

c *Pbwg1.5* was previously defined to an interval between *D2Mit270* and *D2Mit472* [21] (see Figure 1).

Likewise, we were able to confirm the presence of the *Pbwg1.3* QTL affecting total body length that was previously revealed by interval mapping with an F_2_ intercross population between the B6.Cg-*Pbwg1* original congenic and B6 strains [19]. *Pbwg1.3* was physically defined to an 8.0-Mb interval between *D2Mit433* (57.3) and *D2Mit205* (65.3) (Table 4).

In the B6×SR1 intercross, a QTL for which the wild-derived allele decreased inguinal and gonadal fat pad weights was clearly identified. In the B6×SR21 intercross, however, the presence of the obesity QTL was ambiguous, because *P* values for diplotype comparisons marginally exceeded the nominal 5% level but did not reach the Bonferroni-corrected 5% level. Previously, the *Pbwg1.5* QTL for resistance to obesity was physically mapped to an interval between *D2Mit270* (52.9) and *D2Mit472* (61.5) [21] (see Figure 1). Therefore, we were able to confirm the presence of *Pbwg1.5* in this study and localize it to a 2.1-Mb interval between *D2Mit123* (59.4) and *D2Mit472* (61.5) (Table 4).

### Exome Sequencing

Since no sequence data have so far been reported for the Philippine wild *castaneus* mice used in this study, we performed sequencing of 2,205 exons for 153 genes on the 44-Mb original congenic region of chromosome 2. According to RefSeq mm9, target bases for the exons were 767,440 bp. The NimbleGen sequence capture covered 97.1% of the target bases, i.e., 745,515 bp. Individual sequence coverage was 11.2 fold on average and ranged from 3 to 36 fold. As expected, some kinds of sequence variants, such as SNPs and indels, were observed in most genes derived from the wild mouse (Table S5). In total, 840 sSNPs and 334 nsSNPs were identified. Nine deletions and 10 insertions were detected in 13 genes. In addition, five nonsense mutations were identified within three genes. On the QTL regions narrowed by the above intersubspecific subcongenic intercross analyses, many SNPs and a few indels were identified, but no nonsense mutation was detected (Tables 5 and S7).

**Table 5:** Variants detected by exome sequencing of genes on the genomic regions harboring the growth QTL *Pbwg1.12* and the obesity QTL *Pbwg1.5*, prioritization of candidate genes and damage of protein functions caused by nsSNPs found in the candidate genes.

	Position (bp)		Number of variants^a^				Candidate gene ranking^b^		Damage to protein function^c^	
Gene symbol	Start	End	sSNP	nsSNP	Deletion	Insertion	Body weight	Obesity	SIFT	PolyPhen-2
*Dapl1*	59322709	59343075	1	0	0	0	NA			
*Tanc1*	59450100	59684206	21	4	0	1	NA			
*Wdsub1*	59690423	59720663	6	1	0	0	NA			
*Baz2b*	59737419	59963797	15	6	1	0	NA			
*March7*	60047992	60086442	6	0	0	0	NA			
*Cd302*	60090049	60122475	1	0	1	0	NA			
*Ly75*	60131816	60221288	27	9	0	0	NA	1	Tolerated	Benign
*Pla2r1*	60257095	60391318	18	8	0	0	NA			
*Itgb6*	60436349	60511750	11	3	0	0	NA	2	Affected	Benign
*Rbms1*	60590009	60801261	2	0	0	0	NA			
*Tank*	61416642	61492224	1	5	0	0	NA			
*Psmd14*	61549750	61638433	1	0	0	0	NA			
*Tbr1*	61642509	61652170	2	1	0	0		NA		
*Slc4a10*	61884596	62164800	6	0	0	0		NA		
*Dpp4*	62168131	62250288	6	0	0	0		NA		
*Gcg*	62312586	62321710	0	1	0	0	1	NA	Tolerated	Benign
*Fap*	62339001	62412078	2	2	0	0		NA		
*Ifih1*	62433849	62484312	17	5	0	0		NA		
*Gca*	62502383	62532166	3	1	0	0		NA		
*Kcnh7*	62541002	63022344	6	1	0	0		NA		
*Fign*	63815417	63936064	4	1	0	0		NA		
*Grb14*	64750539	64860823	7	2	0	0	2	NA	Tolerated	Benign
*Cobll1*	64926395	65076683	14	18	0	0		NA		

a sSNP, synonymous SNP; nsSNP, nonsynonymous SNP; NA, not applicable because the QTL in question was not located on the region including the genes.

b The top two genes were prioritized as candidate genes for growth and obesity QTLs by the web-based software program Endevour [25].

c Damage caused by nsSNPs was investigated for the ranked genes by two software programs, SIFT [26] and PolyPhen-2 [27].

### Candidate Gene Search

As shown in Table 5, there were 11 genes on the 3.8-Mb region between *D2Mit472* and *D2Mit205*, where the growth QTL *Pbwg1.12* was located. Using training genes related to body weight (Table S1), Endevour prioritized *Gcg* (glucagon) and *Grb14* (growth factor receptor-bound protein 14) as the top two candidate genes for *Pbwg1.12*. *Gcg* had one nsSNP and *Grb14* had two nsSNPs. Both SIFT and PolyPhen-2 predicted that none of these nsSNPs inflicted possible damage on protein functions (Table 5).

On the 2.1-Mb region between *D2Mit123* and *D2Mit472* harboring the obesity QTL *Pbwg1.5*, 12 genes were located (Table 5). Endevour ranked *Ly75* (lymphocyte antigen 75) and *Itgb6* (integrin beta 6) as the top two candidate genes for *Pbwg1.5* using training genes related to obesity (Table S1). *Ly75* had nine nsSNPs and these were predicted to have no affect on protein function. In contrast, *Itgb6* had three nsSNPs. PolyPhen-2 predicted that none of the three nsSNPs caused possible damage to protein function, whereas SIFT predicted that one of them, i.e. A>C at the position of 2:60491216 leading to amino acid substitution of Sel302Ala, is harmful to protein function. This nsSNP has previously been reported as dbSNP rs28025203.

## Discussion

Previously we discovered the *Pbwg1.12* QTL for growth [9], the *Pbwg1.3* QTL for body length [19] and the *Pbwg1.5* QTL for obesity [21] from an untapped resource of wild *M. m. castaneus* mice caught in the Philippines. In this study, we were able to confirm the presence of these three QTLs by intersubspecific intercross analyses using four newly or previously constructed subcongenic strains with overlapping and/or non-overlapping genomic intervals. The unique effects of the wild-derived QTL allele at the QTLs revealed in previous studies [9], [19], [21] were duplicated in the present independent study. That is, this allele uniquely enhanced growth at *Pbwg1.12* and increased body length at *Pbwg1.3*, despite the fact that the wild mouse has approximately 60% of the body weight of B6 [16], whereas it decreased fat weight at *Pbwg1.5*. Furthermore, we were able to reduce the genomic interval harboring *Pbwg1.12* from 8.9 Mb [9] to 3.8 Mb in length and to narrow the interval of *Pbwg1.5* from 8.8 Mb [21] to 2.1 Mb. Although *Pbwg1.3* was previously localized to a 20-Mb confidence interval [19], it was physically mapped to an 8.0-Mb interval in this study.

Although *Pbwg1.3* and *Pbwg1.5* exerted phenotypic effects on both sexes, *Pbwg1.12* exhibited male-specific effect on body weight. In our previous study [9], the sex-specificity of *Pbwg1.12* on chromosome 2 was not tested because the sample size was small. Sex-specific QTLs for body weight are revealed on different chromosomal regions in our previous genome-wide QTL analysis [18] and in different mouse crosses [5]. In addition, sex-specific QTLs have commonly been observed in different quantitative traits of mice and other species, as mentioned previously [16], [18]. However, the molecular mechanisms underlying sex-specific QTLs remain unclear. It is reported that androgen control of growth hormone secretion induces male-specific gene expression in the liver of mice [28]. We thus consider that the male-specific effect of *Pbwg1.12* may be mediated by sex hormones through male-specific expression of the causative gene of *Pbwg1.12*.

According to MGD [5], several QTLs affecting growth, body length and obesity were previously mapped to mouse chromosome 2 regions that are overlapped with our 8.0-Mb region between *D2Mit443* (57.3) and *D2Mit205* (65.3). Since the previous QTLs were mapped by genome-wide QTL analyses, confidence intervals of the QTLs are generally very large, spanning approximately 100 Mb or more. Thereafter, few map positions of QTLs have been determined physically, with a few exceptions. Four growth and obesity QTLs, named *Wg2a*-*Wg2d*, have been fine-mapped to the interval from *D2Ucd15* (74.7) to *D2Mit196* (160.5) by phenotypic analyses of subcongenic strains that possess the introgressed regions of the CAST/EiJ strain established from wild *M. m. castaneus* mice on the B6 genetic background [10]. However, this interval is outside of our QTL regions. The *Nidd5* QTL affecting adiposity has been fine-mapped by phenotypic analysis of congenic strains with donor regions derived from the BALB/cA strain on the genetic background of the obese/diabetic TSOD strain, and *Acvr1c* encoding activin receptor-like kinase 7 at the position of 58.1 Mb was very recently identified as a responsible gene for *Nidd5* [29]. The obese/diabetic TSOD strain unexpectedly has the wild-type allele at *Acvr1c*, whereas the normal BALB/cA strain has a nonsense mutation resulting in decreased fat mass phenotype [29]. In contrast, our exome sequencing analysis indicated that the *Acvr1c* gene derived from our wild *castaneus* mouse had neither nsSNPs nor nonsense mutations. Furthermore, *Acvr1c* lies outside the interval containing our *Pbwg1.12* for increased body weight and *Pbwg1.5* for decreased fat weight. *Acvr1c* is thus unlikely to be a candidate gene for our QTLs. In addition, as no nonsense mutation was identified for any of the genes located in the QTL interval, this kind of mutation could not become a sequence variant causing the differences in body weight and fat weight shown in this study.

In this study, exome sequencing and candidate gene prioritization strongly suggested that *Gcg* and *Grb14* are putative candidate genes for the *Pbwg1.12* QTL for enhanced growth. *Gcg* encodes proglucagon, a precursor of glucagon, glucagon-like peptide-1 (GLP-1) and several other components. Glucagon is generated in pancreatic α-cells and GLP-1 is yielded in intestinal L-cells, and these peptides paly key roles in glucose metabolism and homeostasis [30]. Mice lacking glucagon and GLP-1 are born normally without gross abnormalities and display α-cell hyperplasia and increased body weight [31]. It has very recently been revealed in rats fed a high-fat diet that hypothalamic glucagon signaling can suppress hepatic glucose production, suggesting that hypothalamic glucagon resistance may contribute to the hyperglycemia observed in obesity and diabetes [32]. *Grb14* encodes an adaptor protein belonging to the GRB7 family and it plays an important role in receptor-tyrosine kinase signaling pathways and insulin signaling [33]. *Grb14* knockout mice are born normally, show a small reduction in body weight and exhibit improved glucose homeostasis and enhanced insulin signaling in the liver and skeletal muscle [34]. Judging from the phenotypic similarity between *Pbwg1.12* and knockout mice, *Gcg* is very likely to become a candidate gene for *Pbwg1.12*, although further studies such as pancreatic islet characterization and *Gcg* expression analysis will be needed in our subcongenic mice.

For the obesity *Pbwg1.5* QTL, *Ly75* and *Itgb6* were suggested to be putative candidate genes in this study. *Ly75* encodes DEC-205, a 205-kD integral membrane protein homologous to the macrophage mannose receptor, and DEC-205 is a novel endocytic receptor used by dendritic cells and thymic epithelial cells to direct captured antigens from the extracellular space to a specialized antigen-processing compartment [35]. *Ly75* knockout mice exhibit abnormalities in CD8-positive T cell morphology and cytotoxic T cell physiology [36]. *Itgb6* encodes the integrin β6 subunit, a member of the integrin family. This subunit heterodimerizes with the αv subunit to bind and/or activate latent transforming growth factor β. The expression of αvβ6 integrin is largely restricted to a subset of epithelial cells [37], [38]. *Itgb6* knockout mice are born and grow normally but exhibit juvenile baldness associated with macrophage infiltration of the skin and accumulation of activated lymphocytes around conducting airways in the lungs, suggesting that alterations in this integrin may contribute to the development of inflammatory diseases of epithelial organs including the skin, lungs and kidney [39]. A previous microarray analysis revealed that 259 genes are differentially expressed in the liver between SM/J and LG/J mouse strains fed a high-fat diet, where SM/J is more responsive than LG/J for many obesity and diabetes traits. Most of these genes are associated with immune function, and 62 genes are located within intervals of QTLs previously mapped for obesity, diabetes and related traits [40]. High-fat diets are known to trigger an immune response through inflammation in many organs and tissues such as the liver and adipose tissue [41], [42]. Hence, the genes associated with immune function can become candidate genes for obesity and related QTLs. Therefore, the *Ly75* and *Itgb6* genes with immune function may be good candidate genes for our *Pbwg1.5* that shows prevention of obesity when mice are fed both low-fat standard and high-fat diets [21].

The *Itgb6* gene on the *Pbwg1.5* region derived from a wild *castaneus* mouse caught in the Philippines had the nsSNP of g.2:60491216A>C, leading to amino acid substitution of Sel302Ala that was predicted to be harmful to protein function by SIFT but not by PolyPhen-2. In fact, the Sel residue is conserved among many mammals including humans, dogs, bovines, horses and rats [43]. In mice, both Sel and Ala residues are segregating among common inbred strains and also among wild-derived inbred strains [5]. It is noteworthy that the CAST/EiJ strain established from wild *castaneus* mice in Thailand has the same base substitution (C: Ala) as that of our wild *castaneus* mouse in the Philippines. In addition, QTLs for obesity and related traits identified from CAST/EiJ and other strains have so far not been fine-mapped to the *Pbwg1.5* region on mouse chromosome 2, as discussed earlier. These facts thus suggest that the A>C nsSNP might not act as a sequence variant causing our phenotypic variation.

Next-generation sequencing of 13 classical inbred mouse strains and four wild-derived inbred strains has recently revealed that QTLs with small effects on 100 phenotypes of disease and physiological traits, which were identified in more than 2,000 heterogeneous stock mice, are more likely to arise from intergenic sequence variants lying outside genes and are less likely to arise from nsSNPs and structural variants (indels, inversions, copy number gains and others) lying within genes. In contrast, it has been shown that QTLs with large effects are more likely to arise from structural variants and are less likely to arise from intergenic variants [23], [44]. We therefore consider that, since our QTLs have small effects on growth and obesity, their causative variants may be intergenic variants rather than nsSNPs and structural variants identified by exome sequencing in this study. As the next step, we will need to perform expression analysis of the four putative candidate genes searched in this study. Gene expression results will provide information helpful for identifying causative genes and further causative variants underlying our QTLs on chromosome 2.

In conclusion, by analysis using intersubspecific subcongenic intercrosses, we precisely fine-mapped three unique QTLs for enhanced growth, prevention of obesity and increased body length, which were discovered from a wild *M. m. castaneus* mouse, to small genomic intervals ranging from 2.1 to 8.0 Mb on mouse chromosome 2. By combined analysis of exome sequencing and bioinformatics, we identified four genes as putative candidate genes for the unique growth and obesity QTLs. We furthermore predicted that nsSNPs found in the candidate genes would not be harmful to protein functions.

## Supporting Information

Figure S1 **Two PCR-RFLP markers on mouse chromosome 2 developed in this study.** (A) The *rs13476521* PCR-RFLP marker was constructed on the basis of the *rs13476521* SNP located at 58,131,026 bp on the *Cytip* gene. B6 has the nucleotide base T and our exome sequencing revealed that our wild *castaneus* mouse has the base C being the same as that of CAST/EiJ. A pair of primers, 5′-CCTGGGGGAATGGATAAAGT-3′ and CCTGACTCGGACACTGGAAT, amplified a 364-bp fragment including this SNP. The restriction enzyme *EcoR*V cut the 364-bp fragment derived from B6 in two (195 and 169 bp), whereas it did not cut the 364-bp fragment derived from the wild mouse. (B) The *rs48690987* PCR-RFLP marker was developed on the basis of the *rs48690987* SNP at 62,606,356 bp on the *Ifih1* gene. B6 has the nucleotide base T, whereas our wild mouse has the base C being the same as that of CAST/EiJ. A pair of primers, AAATTCATCCGTTTCGTCCA and GGATAGTTTTCTGCCCTTTGC, amplified a 306-bp fragment. The enzyme *EcoT22*I generated two B6-derived fragments (160 and 146 bp), whereas it did not cut a wild-derived fragment. PCR was performed as described previously [19], and 2.0–2.5% agarose gels were used for electrophoresis.
(TIF)

Table S1 **Training genes used in prioritization of candidate genes.**
(XLSX)

Table S2 **Additive and dominance diplotype effects for body weight and weight gain in the F_2_ populations obtained from B6×SR2 and B6×SR12 intercrosses.**
(XLSX)

Table S3 **Organ weight not adjusted for body weight at 14 weeks of age, and additive and dominance diplotype effects for organ weight in the F_2_ populations obtained from B6×SR1 and B6×SR21 intercrosses.**
(XLSX)

Table S4 **Measurements of body composition traits not adjusted for body weight at 14 weeks of age, and additive and dominance diplotype effects for body composition traits in the F_2_ populations obtained from B6×SR2 and B6×SR12 intercrosses.**
(XLSX)

Table S5 **Organ weight adjusted for body weight at 14 weeks of age, and additive and dominance diplotype effects for organ weight in the F_2_ populations obtained from B6×SR1 and B6×SR21 intercrosses.**
(XLSX)

Table S6 **Measurements of body composition traits adjusted for body weight at 14 weeks of age, and additive and dominance diplotype effects for body composition traits in the F_2_ populations obtained from B6×SR2 and B6×SR12 intercrosses.**
(XLSX)

Table S7 **Summary of variants identified by exome sequencing of 153 genes located on the original congenic region between** ***D2Mit33*** **and** ***D2Mit38*** **on mouse chromosome 2.**
(XLSX)
