# Supplementary figures and images for: Fine Mapping and Candidate Gene Search of Quantitative Trait Loci for Growth and Obesity Using Mouse Intersubspecific Subcongenic Intercrosses and Exome Sequencing
Source: PLoS One. 2014 Nov 14;9(11):e113233. doi: 10.1371/journal.pone.0113233 (PMC4232600; doi:10.1371/journal.pone.0113233)

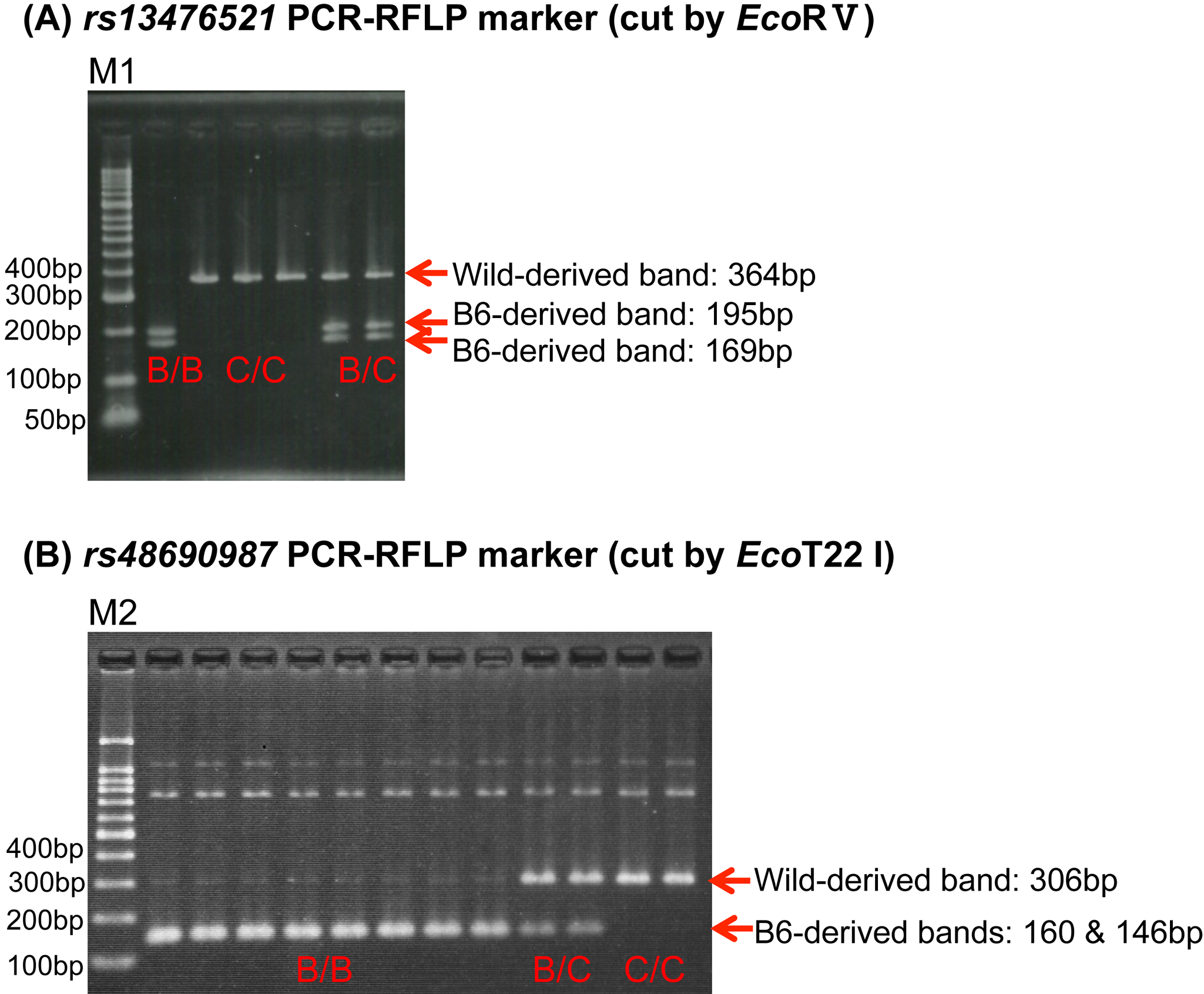

Supplement: Figure S1 — Two PCR-RFLP markers on mouse chromosome 2 developed in this study. (A) The rs13476521 PCR-RFLP marker was constructed on the basis of the rs13476521 SNP located at 58,131,026 bp on the Cytip gene. B6 has the nucleotide base T and our exome sequencing revealed that our wild castaneus mouse has the base C being the same as that of CAST/EiJ. A pair of primers, 5′-CCTGGGGGAATGGATAAAGT-3′ and CCTGACTCGGACACTGGAAT, amplified a 364-bp fragment including this SNP. The restriction enzyme EcoRV cut the 364-bp fragment derived from B6 in two (195 and 169 bp), whereas it did not cut the 364-bp fragment derived from the wild mouse. (B) The rs48690987 PCR-RFLP marker was developed on the basis of the rs48690987 SNP at 62,606,356 bp on the Ifih1 gene. B6 has the nucleotide base T, whereas our wild mouse has the base C being the same as that of CAST/EiJ. A pair of primers, AAATTCATCCGTTTCGTCCA and GGATAGTTTTCTGCCCTTTGC, amplified a 306-bp fragment. The enzyme EcoT22I generated two B6-derived fragments (160 and 146 bp), whereas it did not cut a wild-derived fragment. PCR was performed as described previously [19], and 2.0–2.5% agarose gels were used for electrophoresis. (TIF) [file pone.0113233.s001.tif]
